# Supplementary material for: A Simple and Label-Free Detection of As3+ using 3-nitro-L-tyrosine as an As3+-chelating Ligand
Source: Sensors (Basel). 2019 Jun 27;19(13):2857. doi: 10.3390/s19132857 (PMC6651597; doi:10.3390/s19132857)
Supplement: Supplementary file 1 [file sensors-19-02857-s001.pdf]

Supporting Information

A Simple and Label-free Detection of As^{3+} using 3-nitro-L-tyrosine as a As^{3+}-chelating ligand

Jin-Ho Park [1,2], Gyuho Yeom [1], Donggu Hong [1], Eun-Jung Jo [1], Chin-Ju Park [1] and Min-Gon Kim [1,*]

[1]Department of Chemistry, Gwangju Institute of Science and Technology (GIST), 123 Cheomdangwagi-ro, Buk-Gu, Gwangju 61005, Republic of Korea; jhp1223@gist.ac.kr (J.-H.P.); ygh0727@gist.ac.kr (G.Y.); inkohdg@gist.ac.kr (D.H.); jej@gist.ac.kr (E.-J.J.); cjpark@gist.ac.kr (C.-J.P.)

[2]Center for Systems Biology, Massachusetts General Hospital, Harvard Medical School, Boston, MA 02114, USA; jpark93@mgh.harvard.edu (J.-H.P.)

*****Correspondence: mkim@gist.ac.kr (M.-G.K.); Tel.: +82-62-3330; Fax: +82-62-2887

Table S1. Parameter values obtained via ITC measurement

Model	Parameter	Value
Blank (constant)	Blank (μJ)	16.9669
Independent	K_a (M^{-1})	2.13E+05
	n	0.640
	△H (kJ/mol)	5.787
	K_d (M)	4.70E-06
	S (J/mol·K)	121.4

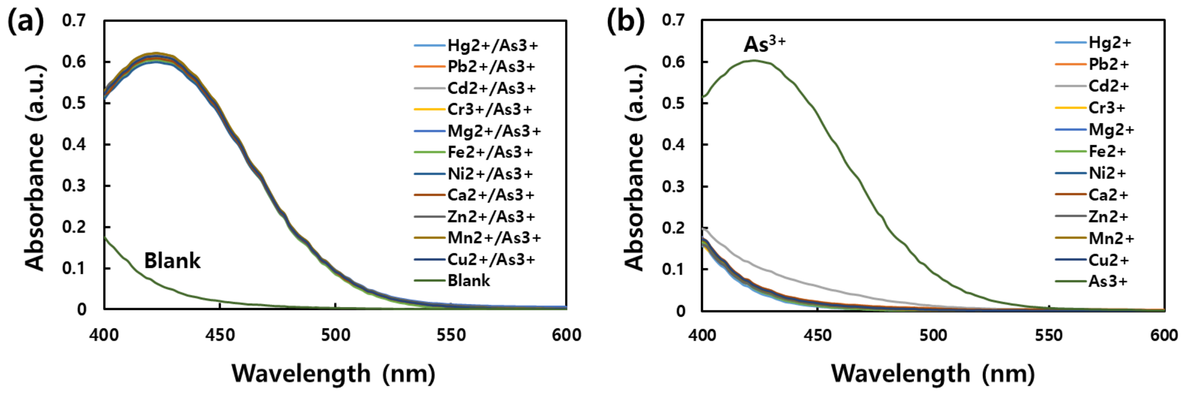

Figure S1. Absorption spectra of mixtures of metal ions and N-Tyr (a) with or (b) without As^{3+}.

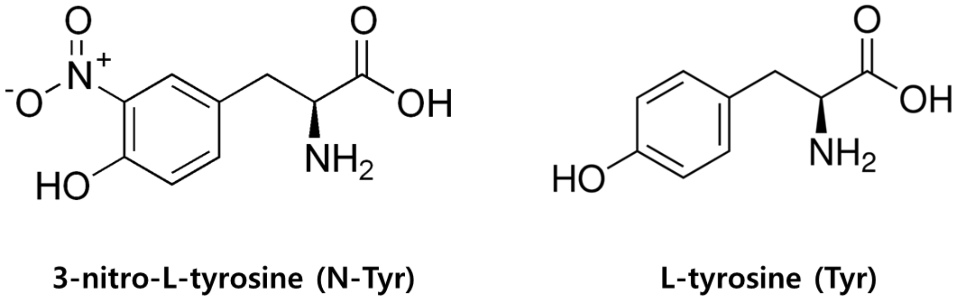

Figure S2. Chemical structure of N-Tyr and Tyr. A NO_2 group exists in chemical structure of N-Tyr.

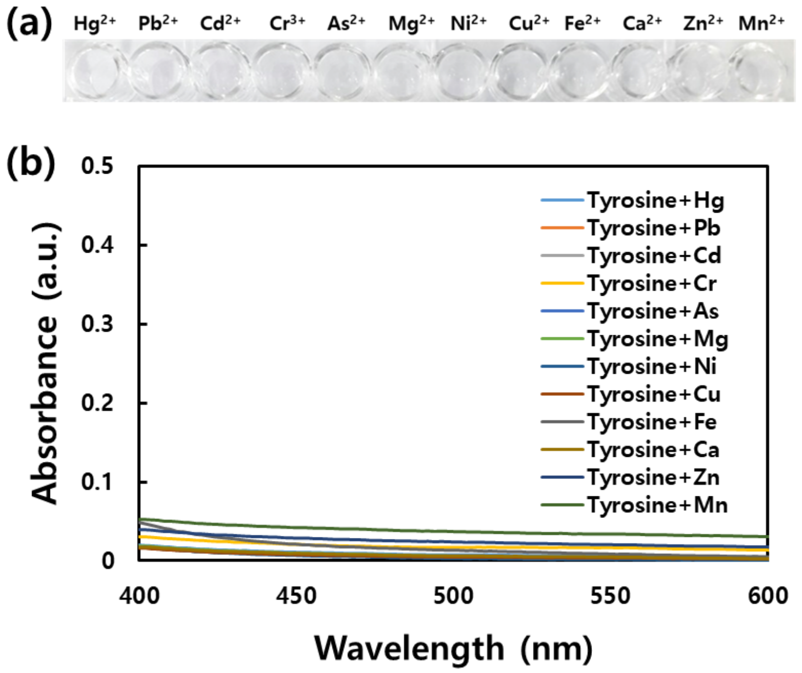

Figure S3. The reaction between various metal ions and Tyr. (a) Images of the mixtures and (b) the corresponding absorption spectra are shown.

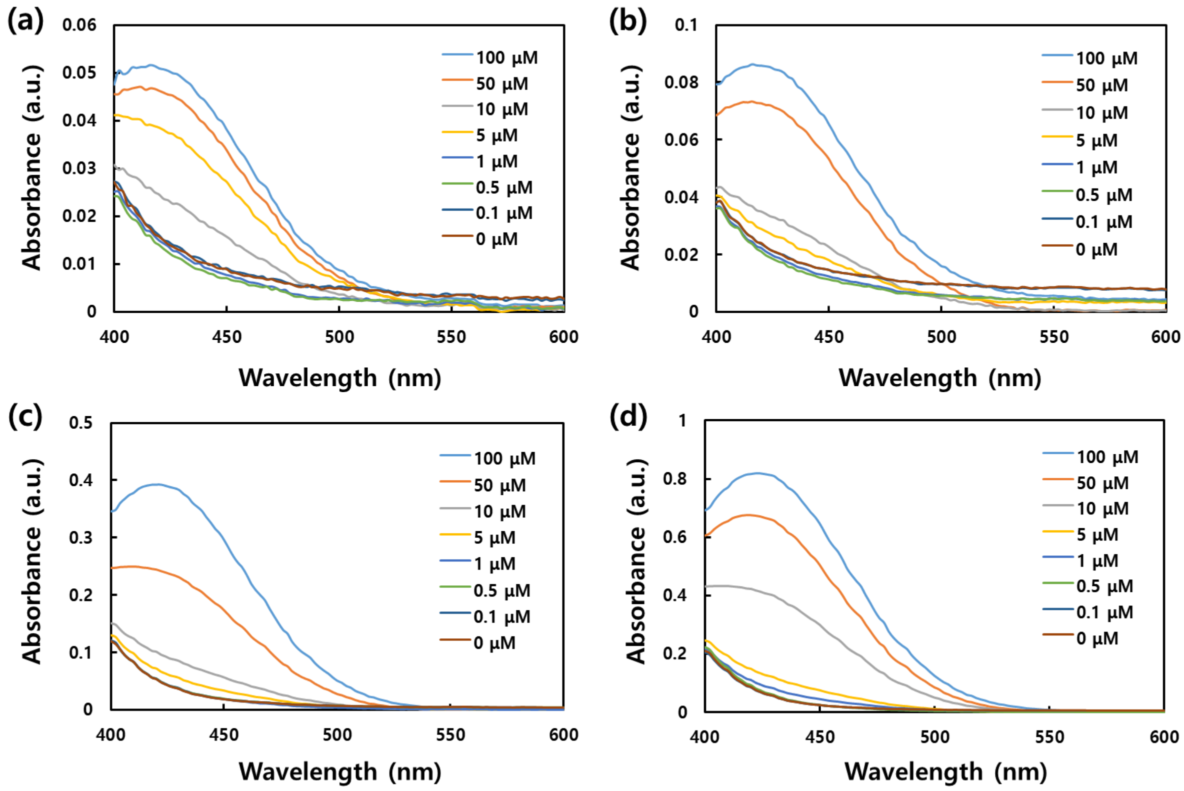

Figure S4. Absorption spectra depending on As^{3+} with different concentrations of N-Tyr, (a) 50 μM, (b) 100 μM, (c) 500 μM and (d) 1,000 μM.

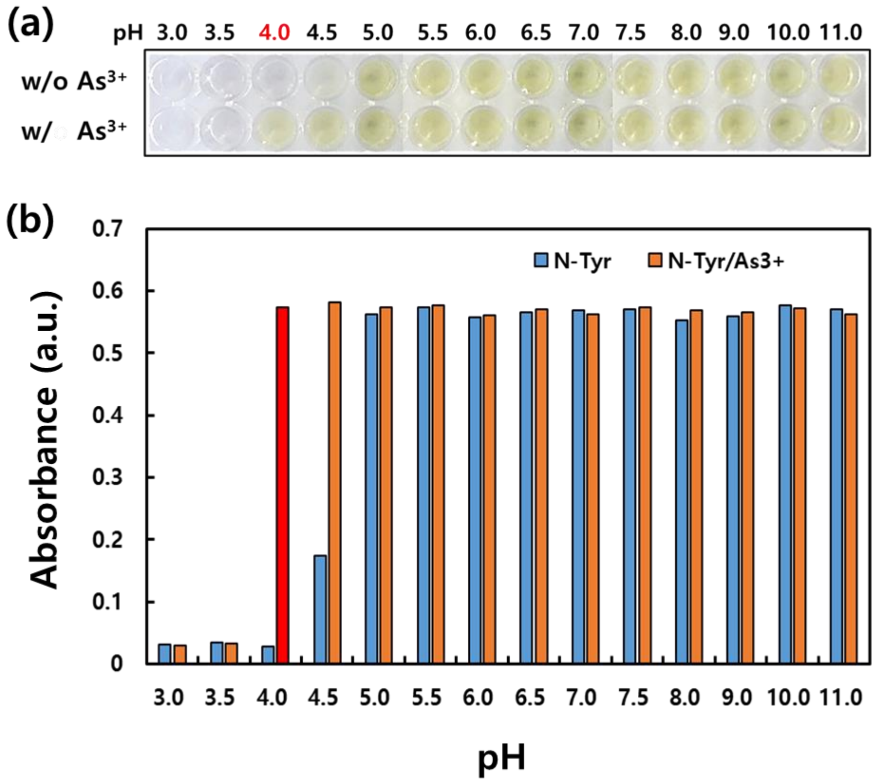

Figure S5. Various pHs were tested with two experimental conditions, N-Tyr alone and As^{3+}/N-Tyr mixture. A selective yellow color with As^{3+} was appeared under pH 4.0. In this test, 0.5 mM N-Tyr and 1 mM As^{3+} were used.

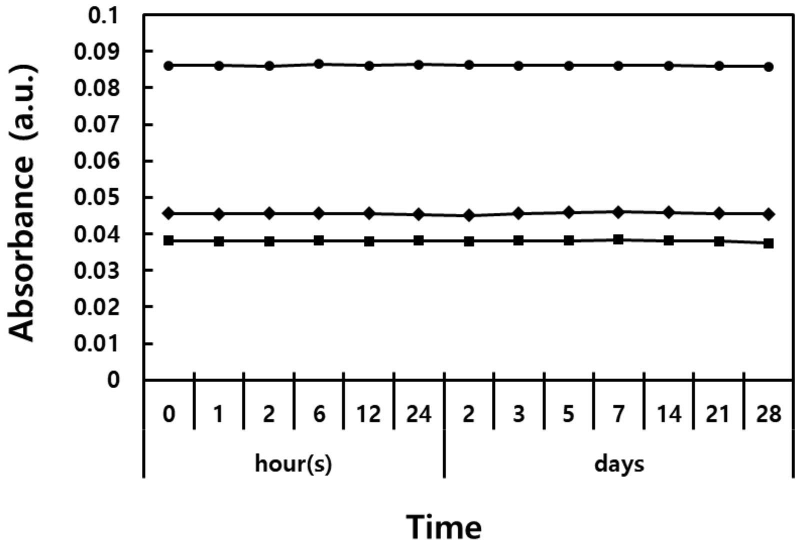

Figure S6. Stability of the As^{3+}/N-Tyr reaction was checked. Three different A^{3+} concentrations, e.g., 0.1 (square), 1 (diamond), and 10 μM (circle), were reacted with 0.5 mM N-Tyr then, stayed at RT condition for 28 days. Almost same absorbance degrees were obtained from each measurement.
